# A novel stable isotope tracer method to simultaneously quantify skeletal muscle protein synthesis and breakdown

**DOI:** 10.1016/j.metop.2020.100022

**Published:** 2020-01-07

**Authors:** Hannah Crossland, Kenneth Smith, Philip J. Atherton, Daniel J. Wilkinson

**Affiliations:** MRC-ARUK Centre for Musculoskeletal Ageing Research, National Institute for Health Research (NIHR) Biomedical Research Centre (BRC), Clinical, Metabolic and Molecular Physiology, University of Nottingham, Royal Derby Hospital, Derby, UK

**Keywords:** Skeletal muscle, Protein synthesis, Protein breakdown, Stable isotope tracers

## Abstract

**Background/aims:**

Methodological challenges have been associated with the dynamic measurement of muscle protein breakdown (MPB), as have the measurement of both muscle protein synthesis (MPS) and MPB within the same experiment. Our aim was to use the transmethylation properties of methionine as proof-of-concept to measure rates of MPB via its methylation of histidine within skeletal muscle myofibrillar proteins, whilst simultaneously utilising methionine incorporation into bound protein to measure MPS.

**Results:**

During the synthesis measurement period, incorporation of methyl[D_3_]-^13^C-methionine into cellular protein in C2C12 myotubes was observed (representative of MPS), alongside an increase in the appearance of methyl[D_3_]-methylhistidine into the media following methylation of histidine (representative of MPB). For further validation of this approach, fractional synthetic rates (FSR) of muscle protein were increased following treatment of the cells with the anabolic factors insulin-like growth factor-1 (IGF-1) and insulin, while dexamethasone expectedly reduced MPS. Conversely, rates of MPB were reduced with IGF-1 and insulin treatments, whereas dexamethasone accelerated MPB.

**Conclusions:**

This is a novel stable isotope tracer approach that permits the dual assessment of muscle cellular protein synthesis and breakdown rates, through the provision of a single methionine amino acid tracer that could be utilised in a wide range of biological settings.

## Highlights

• Measuring protein synthesis and breakdown simultaneously is methodologically challenging.
• The stable isotope tracer methyl[D3]-13C-methionine was able to measure protein synthesis *in vitro.*
• The same tracer could also measure breakdown after its conversion to methyl[D3]-methylhistidine.
• This method could be widely used by researchers within the field of musculoskeletal research.

## 1 Introduction

The use of stable isotope labelled compounds in musculoskeletal research, through dynamic measurement of rates of metabolism and turnover of proteins across a range of conditions and interventions, has allowed us to gain crucial insights into fuel and protein metabolism in health and disease [1,2]. Studying protein turnover and its relation to the regulation of muscle mass in terms of both hypertrophy (muscle growth) and atrophy (muscle loss) is of key importance for understanding the regulation of metabolic health and the role of skeletal muscle in health and disease. For measurement of muscle protein synthesis (MPS) using stable isotope tracers, these techniques have been extensively developed over the years, from the use of direct incorporation measures, using precursor-product methods, to the use of arterio-venous (A-V) balance across a tissue bed [1].

However, method development for the measurement of muscle protein breakdown (MPB) has been associated with greater challenges, due to the complex nature of the study design employed (multiple tracer boluses or constant infusions), the complex mathematical assumptions required, as well as the assumption that there is minimal recycling of the amino acid (AA) tracer back into bound protein pool [3,4]. Hence, the relative paucity of information relating to control of protein breakdown in physiological systems and conditions. One primary method for assessing dynamic MPB involves infusion/incubation of a stable isotope labelled amino acid and measuring its rate of appearance (via dilution of the tracer) from tissues or cells [5,6]. These models have provided important physiological insights into musculoskeletal protein metabolism, although there are certain limitations associated with the technique, such as the assumption of steady state, and underestimation of MPB due to the possibility of reutilization of amino acids for MPS [7].

Another approach to assess MPB involves measurement of 3-methylhistidine, a post-translationally methylated form of histidine found in myofibrillar proteins. Once released from the peptide during protein breakdown, methylhistidine cannot be reincorporated into proteins, and is excreted from the body in the urine. The urinary excretion of methylhistidine has been frequently used as a proxy measure of whole body and skeletal muscle protein breakdown [8,9], although there has been debate as to how accurately this reflects muscle as methylhistidine can be formed in other proteins in the body i.e. the gut [10] (although the majority is believed to be contained in actin and myosin filaments [11]).

The aim of the present study was to develop a novel stable isotope tracer approach that overcomes some the issues associated with current protein breakdown measures, and was able to measure MPS and MPB within the same experiment utilising a single isotopically labelled amino acid tracer. Using methyl[D_3_]-^13^C-methionine, we have developed this proof-of-concept method, utilising murine C2C12 myotubes *in vitro*, performing a time-course study for both MPS and MPB. We measured the incorporation of methyl[D_3_]-^13^C-methionine into protein to determine protein synthesis and measured the rate of appearance of methyl[D_3_]-histidine, following the transfer of the tri-deuterated methyl group of methyl[D_3_]-^13^C-methionine, via [methyl-D_3_]-S-adenosyl-^13^C-methionine onto histidine residues in myosin and actin, as a measure of proteolysis of myofibrillar proteins. Furthermore, we have validated the approach using interventions that are known to either positively (IGF-1/insulin) and/or negatively (Dexamethasone) regulate MPS and MPB.

## 2 Materials and methods

### 2.1 C2C12 cell culture

Murine C2C12 cells (ECASS, Salisbury, UK) were maintained in Dulbecco’s Modified Eagle Medium (DMEM; Thermo Fisher Scientific) containing 10% (v/v) fetal bovine serum (FBS), 1% (v/v) antibiotic-antimycotic solution and 4 mM l-glutamine (all from Sigma-Aldrich, UK) and kept in a 37 °C incubator with 5% CO_2_. Myoblasts were seeded onto collagen-coated (Type I from rat tail; Sigma-Aldrich, UK) six-well multidishes (Nunclon™ Delta; Thermo Fisher Scientific) and when confluency reached ∼90%, differentiation was induced by changing to DMEM containing 2% (v/v) horse serum, 1% (v/v) antibiotic-antimycotic solution and 4 mM l-glutamine (Sigma-Aldrich, UK). The media was changed every 48 h and experiments were performed on days 4–5 post-switch to differentiation media.

### 2.2 Methyl[D_3_]-^13^C-methionine tracer experiments

The theory behind the technique relates to the transmethylation properties provided by the essential AA methionine. Following conversion to S-adenosylmethionine (SAMe) by methionine adenosyltransferase, this compound can act as a methyl donor for a number of compounds [12,13], including the post-translational modification of histidine residues to methylhistidine following peptide formation, principally in actin and myosin in muscle [13].

**Fig. 1:**
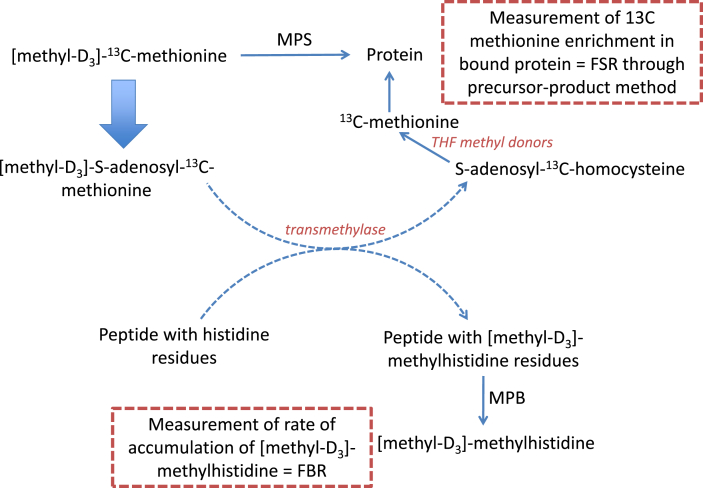
**Schematic representing proposed mechanism of action of [methyl-D_3_]-^13^C-methionine tracer.** Methyl[D_3_]-^13^C-methionine will primarily act as substrate for muscle protein synthesis (MPS) within the cell, however a proportion of the methionine will act as a methyl donor, transferring the methyl[D_3_] group to other compounds, including histidine in the bound protein. Following muscle protein breakdown (MPB), methyl[D_3_]-methylhistidine will be released and its rate of cellular/extracellular accumulation will be representative of the rate of MPB.

The method developed in the present study is based on the theory that provision of methyl[D_3_]-^13^C-methionine will primarily act as substrate for MPS within the cell; however also that a proportion of the methionine will act as a methyl donor, transferring the methyl[D_3_] group to other compounds, including histidine in the bound protein (see Fig. 1). Following MPB, this methyl[D_3_]-methylhistidine will be released and its rate of cellular/extracellular accumulation will be representative of the rate of MPB. Moreover, through the addition of the ^13^C label, the methyl[D_3_]-^13^C-methionine that is not utilised as a methyl donor (and potentially that which is too, as homocysteine can be methylated back to methionine via folate intermediates) will be incorporated into new protein, meaning that we can monitor the rate of cellular protein synthesis by measuring the rate of incorporation of this D_3_^13^C-methionine into protein. Similarly, we could have utilised a methionine tracer, containing only a deuterated Methyl moiety.

Initial experiments aimed to assess the time course of methyl[D_3_]-^13^C-methionine incorporation into cellular protein, and the subsequent disappearance from cells (and appearance in media) in the form of D_3_-3-methylhistidine. For MPS measures, methyl[D_3_]-^13^C-methionine was added into the media (to achieve an enrichment of ∼15 atom percent excess (APE)) at the point of the media change on day 4 post differentiation. Cells were incubated for 4, 6, 8, 24 and 48 h, after which the media was collected, and cells were harvested in 0.2 mol L-1 perchloric acid (PCA) for GC-MS analysis (see below). For MPB measures, cells were incubated with methyl[D3]-13C-methionine (as above) for 24 h, after which cells were washed with Hank’s balanced salt solution (HBSS) and fresh media (without tracer) replaced. Cells and media were collected after 4, 6, 8, 24 and 48 h. All experiments were repeated twice and performed using n = 4–6 well replicates each time.

For determining whether the method can quantifiably detect differences in MPS and MPB under different conditions, cells were incubated with selected compounds known to influence MPS and/or MPB (insulin-like growth factor 1 (IGF-1), 50 ng/ml; insulin, 300 nmol L-1, and dexamethasone (Dex), 1 μmol L-1). For MPS measures, treatments were added at the same time as the tracer, whereas for MPB, treatments were added following the addition of tracer-free media after an incubation period with the tracer. In both cases, samples were harvested after 24 h or 48 h treatment. For measuring protein markers of MPS/MPB following each treatment, cells were harvested in homogenisation buffer (50 mmol L-1 Tris-HCl, pH 7.5, 1 mmol L-1 EDTA, 1 mmol L-1 EGTA, 10 mmol L-1 ß-glycerophosphate, 50 mmol L-1 NaF and complete protease inhibitor cocktail tablet (Roche, West Sussex, UK)) for Western blot analysis (see below).

### *2.*3 GC-MS analysis of methyl[D_3_]-^13^C-methionine

Harvested cells were centrifuged (10,000 rpm, 10 min) and the pellet was washed twice with 70% (v/v) ethanol. The pellet was then hydrolysed overnight at 100 °C in 1 ml of HCl (0.1 mol L-1) with 1  ml H+ dowex resin in ddW. Hydrolysed AA were isolated by passing through dowex columns, eluted into 2 mol L-1 NH4OH and dried down. Labelling (^13^C/D_3_) of protein-bound methionine was determined using GC-MS following MTBSTFA derivatization and single ion monitoring (SIM) of m/z 320, 324. Media samples from each well were also purified via dowex columns and dried down and derivatized in the same way as for the cells. Fractional synthesis rate (FSR) was calculated using the following equation:

FSR (%/h) = -ln(1-(APE_product_/APE_precursor_)/t)×100

where APE_product_ = the change in protein-bound enrichment of methyl[D_3_]-^13^C-methionine, APEprecursor = the average in enrichment of methyl[D_3_]-^13^C-methionine in the media and t = time in hours.

### *2.*4 LC-MS analysis of methyl[D3]-3-methylhistidine

Cell and media samples were processed in the same manner as for MPS measures to the point of being dried down after elution from dowex columns. Labelling of methyl-D_3_-3-methylhistidine was performed using HILIC phase liquid chromatography coupled to ESI-high resolution mass spectrometry (Q-Exactive, Thermo Scientific, Hemel Hempstead, UK) using single ion monitoring (SIM) of m/z 170.09230, 173.11131 for 3-MH and D_3_-3 MH respectively.

Fractional breakdown rate (FBR) was calculated using the following equation:

FBR (%/h) = -ln(1-(APE_precursor_/APE_product_)/t)×100

where APE_product_ = the change in protein-bound enrichment of methyl[D_3_]-3-methylhistidine, APEprecursor = the change in enrichment of methyl[D_3_]-3-methylhistidine in the media and t = time in hours.

### 2.5 Western blot analysis

Cell samples were lysed by repeatedly passing through gel-loading pipette tips. Lysates were centrifuged (13,000 g, 10 min, 4 °C) and protein (10 μg) was loaded onto Criterion XT 12% gels (Bio-Rad, UK) for electrophoresis at 200 V for 1 h. Proteins were transferred to PDVF membranes (100 V, 45 min), then membranes were blocked in 5% (w/v) milk for 1 h at room temperature. Primary antibody incubations (p70 S6K1 Thr389, 1:2000; AKT Ser473, 1:2000; eEF2 Thr56, 1:2000; LC3B, 1:2000; and MAFbx, 1:2000; all primary antibodies were purchased from Cell Signaling) were performed overnight at 4 °C, after which membranes were washed for 3 × 5 min using 1x Tris-buffered saline (TBS)-Tween then incubated in HRP-conjugated anti-rabbit secondary antibody (1:2000 dilution) for 1 h at room temperature. Membranes were subsequently washed a further 3 × 5 min in TBS-Tween, then bands were detected using Chemiluminescent HRP Substrate (Millipore EMD) on a Chemidoc XRS imaging system (Bio-Rad, UK). Intensity of bands were normalised against total protein loading through Coomassie staining of the membrane.

### 2.6 Statistical analysis

Data are presented as mean + SEM and analysed using GraphPad Prism version 7 (La Jolla, USA). For comparing differences between treatments and untreated controls, one-way ANOVA was used, with correction for multiple comparisons using Sidak’s test, with *P* < 0.05 taken as statistically significant

## 3 Results

### 3.1 Time course of methyl[D_3_]-^13^C-methionine incorporation and methyl[D_3_]-3-methylhistidine appearance from cells

**Fig. 2:**
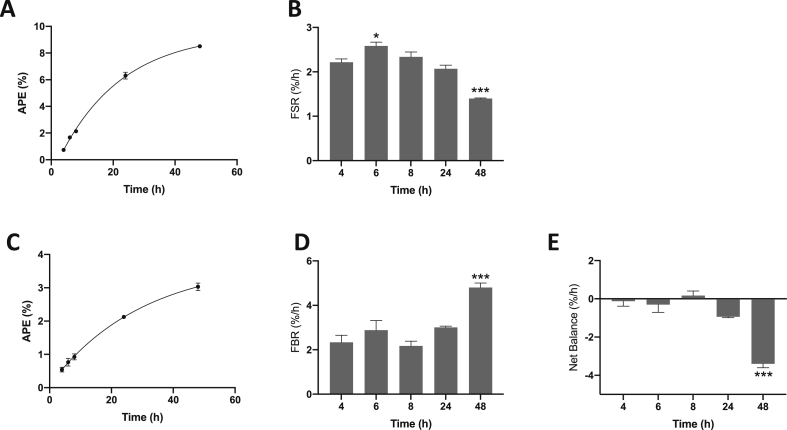
**Time course of [methyl-D_3_]-^13^C-methionine incorporation and cellular release of [methyl-D_3_]-methylhistidine in C2C12 myotubes.** C2C12 myotubes were incubated for various times with [methyl-D_3_]-^13^C-methionine. A & B, changes in enrichment of protein-bound methionine over time, and fractional synthetic rate (FSR) in %/h over time. C & D, changes in enrichment of media [methyl-D_3_]-methylhistidine over time, and fractional breakdown rate (FBR) in %/h over time. E, net balance across time, expressed as %/h. Data are mean ± SEM, n = 4 well replicates for each time point. *; P < 0.05, ***; P < 0.001 vs. 4 h.

Incorporation of methyl[D_3_]-^13^C-methionine into protein in C2C12 myotubes was observed over time in a non-linear manner (Fig. 2A), and by 48 h had reached an enrichment in cellular protein of 8.5 ± 0.1%. MPS rates were higher at 6 h post addition of the tracer (with media change) relative to at 4 h (2.2 ± 0.1%/h at 4 h; 2.6 ± 0.1%/h at 6 h; *P* < 0.05 vs. 4 h), and gradually declining over the next 24 h and by 48 h were lower to that of 4 h (1.4 ± 0.01%/h at 48 h; *P* < 0.001 vs. 4 h; Fig. 2B).

During initial development of the method, we were unable to detect the generation of a fragment containing the ^13^C mass only (*m*+1) via GC-MS, therefore FSR calculations were made using the enrichment from the ^13^C and [D_3_]-containing (*m*+4) compound. It is possible that use of an alternative derivative could generate a fragment containing the ^13^C solely, however similar rates for FSR were observed using the masses chosen.

LC-MS analysis of methyl[D_3_]-3-methylhistidine showed that following an initial incubation period with methyl[D_3_]-^13^C-methionine (and subsequent removal from the media), there was an increase in the appearance of methyl[D_3_]-3-methylhistidine into the cell culture media over time (Fig. 2C), while cellular bound methyl[D_3_]-methylhistidine enrichment decreased. Calculations of FBR showed that over time MPB rates remained relatively constant (between 4 h (2.3 ± 0.3%/h) and 8 h (2.2 ± 0.2%/h)), and increased by 48 h (4.8 ± 0.2%/h; *P* < 0.001 vs. 4 h) post media change (Fig. 2D). Net balance (calculated as MPS - MPB) across the time course remained close to zero (Fig. 2E), but was significantly decreased by 48 h post media change (*P* < 0.001 vs. 4 h).

### *3.2* *Effect of selected treatments on methyl[D_3_]-^13^C-methionine incorporation and methyl[D_3_]-3-methylhistidine appearance from cells*

**Fig. 3:**
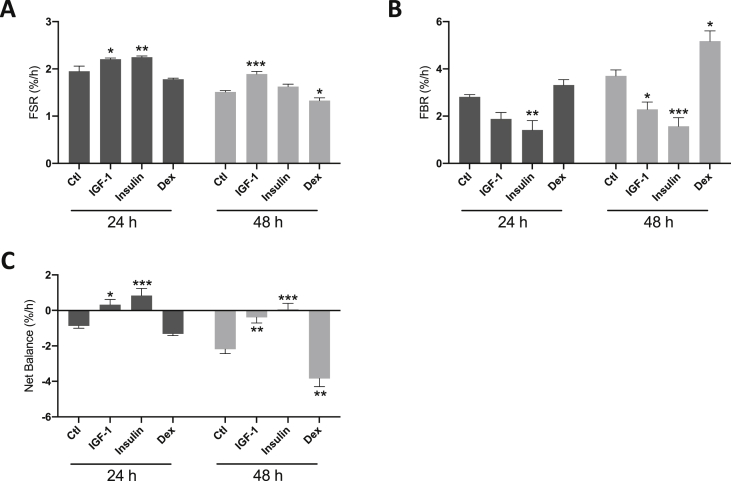
**Effect of selected treatments on [methyl-D_3_]-^13^C-methionine incorporation and cellular release of [methyl-D_3_]-methylhistidine in C2C12 myotubes.** C2C12 myotubes were incubated for 24 h or 48 h with [methyl-D_3_]-^13^C-methionine, and treated with insulin-like growth factor 1 (IGF-1; 50 ng/ml), insulin (300 nmol L^−1^) or dexamethasone (Dex; 1 μmol L^−1^). In breakdown experiments, treatments were added following removal of the tracer after an initial 24 h incubation period. A, changes in fractional synthetic rate (FSR) following IGF-1, insulin or Dex treatments. B, changes in fractional breakdown rate (FBR) following IGF-1, insulin or Dex treatments. C, net balance (%/h) following IGF-1, insulin or Dex treatments. Data are mean ± SEM, n = 6 well replicates for each group. *; *P* < 0.05, **; *P* < 0.01, ***; *P* < 0.001 vs. control.

A 24 h and 48 h period of selected treatments known to influence MPS and/or MPB was undertaken to test the validity of the tracer. Over 24 h, IGF-1 caused a significant increase in MPS (2.2 ± 0.03%/h vs. 1.9 ± 0.1%/h; *P* < 0.05; Fig. 3A) and was still elevated above control by 48 h. Equally, IGF-1 suppressed MPB at 48 h (2.3 ± 0.3%/h vs. 3.7 ± 0.3%/h; *P* < 0.05 vs. control; Fig. 3B). With insulin treatment, the FSR of muscle protein was elevated at 24 h (2.3 ± 0.03%/h; *P* < 0.01; Fig. 3A) compared to control, while at 48 h it was not significantly different from control (Fig. 3A). As expected, there was a marked decrease in MPB at both 24 h and 48 h (1.4 ± 0.4%/h vs. 2.8 ± 0.1%/h at 24 h; *P* < 0.01 vs. control; 1.6 ± 0.1%/h vs. 3.7 ± 0.3%/h at 48 h; *P* < 0.001 vs. control; Fig. 3B). Finally, treatment with the catabolic agent Dex resulted in a significant suppression of MPS at 48 h only (1.3 ± 0.1%/h vs. 1.5 ± 0.03%/h; *P* < 0.05 vs. control; Fig. 3A) as well as increases in MPB (5.2 ± 0.4%/h vs. 3.7 ± 0.3%/h; *P* < 0.05 vs. control; Fig. 3B). Net balance was therefore significantly higher with both insulin and IGF-1 treatments relative to untreated cells (Fig. 3C), and lower than control following 48 h Dex treatment (Fig. 3C; *P* < 0.01 vs. control). These treatments provide proof-of-concept validation that our tracer approach is sensitive to measure changes in both MPB and MPS.

### *3.3* *Effect of selected treatments on total protein content and markers of MPS/MPB*

**Fig. 4:**
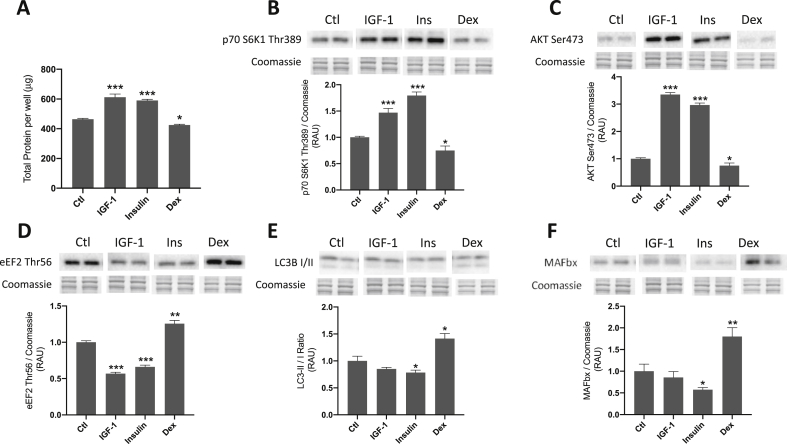
**Effect of selected treatments on total protein and anabolic/catabolic signaling in C2C12 myotubes.** C2C12 myotubes were treated for 24 h with insulin-like growth factor 1 (IGF-1; 50 ng/ml), insulin (300 nmol L-1) or dexamethasone (Dex; 1 μmol L-1). A, total protein per well following IGF-1, insulin or Dex treatments. B–F, changes in phosphorylated p70 S6K1 (Thr389), phosphorylated AKT (Ser473), phosphorylated eEF2 (Thr56), ratio of LC3-II/I and total MAFbx protein following IGF-1, insulin or Dex treatments. Data are mean ± SEM, n = 6 well replicates for each group. *; *P* < 0.05, **; *P* < 0.01, ***; *P* < 0.001 vs. control.

Total protein content and selected protein markers of MPS and/or MPB were measured to further verify the effects of each treatment. Total protein content per well was increased following 24 h IGF-1 and insulin treatments (613 ± 21 μg and 590±8 μg versus 460±5 μg; both *P* < 0.001 vs. control), while protein was decreased after Dex treatment (425±5 μg; *P* < 0.05 vs. control; Fig. 4A).

Phosphorylated p70 S6K1 was significantly elevated by both IGF-1 (1.5 ± 0.08-fold; *P* < 0.001 vs. control) and insulin (1.8 ± 0.07-fold; *P* < 0.001 vs. control) treatments, indicating activation of p70S6K1 activity, whereas Dex caused a reduction in p70 S6K1 Thr389 (0.75 ± 0.09-fold; *P* < 0.05 vs. control; Fig. 4B). Similarly, phosphorylated AKT (Ser473) was significantly increased by both IGF-1 (3.4 ± 0.07-fold; *P* < 0.001 vs. control) and insulin (2.9 ± 0.07-fold; *P* < 0.001 vs. control) treatments (which is indicative of AKT activation), whereas Dex caused a reduction in phosphorylated AKT (0.75 ± 0.09-fold; *P* < 0.05 vs. control; Fig. 4C). Following both 24 h IGF-1 and insulin incubations, phosphorylated eEF2 (Thr56) was significantly decreased (consistent with increased translation elongation) (0.57 ± 0.02-fold; *P* < 0.001 vs. control and 0.66 ± 0.03-fold; *P* < 0.001 vs. control, respectively; Fig. 4D), while eEF2 Thr56 phosphorylation was increased with Dex (1.3 ± 0.04-fold; *P* < 0.01 vs. control).

The ratio of LC3-II to LC3-I protein was unaltered with IGF-1 treatment, but was significantly decreased with insulin (0.8 ± 0.04-fold; *P* < 0.05 vs. control; Fig. 4E), while following Dex treatment, the ratio of LC3-II to LC3-I protein was increased (1.4 ± 0.1-fold; *P* < 0.05 vs. control). Following 24 h IGF-1 incubation, MAFbx protein was unchanged, but with insulin MAFbx protein was significantly decreased (0.6 ± 0.1-fold; *P* < 0.05 vs. control; Fig. 4F), while total MAFbx was increased with Dex (1.8 ± 0.2-fold; *P* < 0.01 vs. control).

## 4 Discussion

While many crucial insights into skeletal muscle protein homeostasis with health and during catabolic conditions such as cachexia and sarcopenia have been gained with the use of stable isotope tracers [[14], [15], [16]], methodological challenges with the measurement of MPB have been faced [7,17]. In this proof-of-concept study, our aim was to use the transmethylation properties of methionine to measure MPB through the methylation of histidine within bound muscle proteins and its subsequent release as labelled methyl[D_3_]-3-methylhistidine, whilst also utilising methionine incorporation into bound protein to measure MPS simultaneously. We were able to demonstrate that the dual labelled tracer methyl[D_3_]-^13^C-methionine could be used to both accurately assess MPS rates in muscle cells *in vitro*, whilst also being used to quantify MPB through LC-MS analysis of methyl[D_3_]-3-methylhistidine release from cellular proteolysis into the media. This provides the first stable isotope tracer technique that can measure both muscle protein synthesis and myofibrillar breakdown rates through the simple provision of a single amino acid tracer.

Development of the use of methyl[D_3_]-^13^C-methionine as a suitable tracer for assessing MPS was achieved by monitoring its incorporation into cellular protein over time (Fig. 2). There was (as expected) a non-linear increase in cellular enrichment of the tracer, with relatively steady rates of MPS over the 48 h period and the highest rates of MPS at 6 h post-media change. The reason for the relative decrease in net balance at 48 h could reflect cellular responses to the media change in terms of substrate depletion (i.e. a relative increase in MPB and decrease in MPS). Nevertheless, these findings indicated that this tracer could be used to accurately estimate MPS *in vitro* similar to our previously established approaches such as ^13^C proline, and D2O [18,19].

Current techniques used to measure MPB includes analysis of 3-MH, a post-translationally methylated form of histidine that arises from degradation of actin and myosin. Measurement of 3-MH can be used to measure myofibrillar proteolysis and thus estimate MPB, since once formed it cannot be further metabolized nor reincorporated into protein [8,9]. In addition, measuring the rate of release of a labelled amino acid from cells represents an alternative method used to quantify MPB. This involves initial incubation with a labelled form of an amino acid that cannot be synthesized or metabolized by muscle (e.g. tyrosine), therefore its release from cellular protein can be used to assess rates of MPB [5]. However, certain problems can arise if labelled amino acids released during MPB are recycled back into cellular protein, which can result in substantial underestimation of MPB rates. Our approach was to test the hypothesis that the methyl[D_3_] group from methyl[D_3_]-^13^C-methionine would be transferred to other compounds, including the histidine residues within bound protein. This would allow us to measure the appearance and rate of release of methyl[D_3_]-methylhistidine following muscle proteolysis. A time course following an initial incubation period with the tracer, and subsequent removal, demonstrated non-linear increases in methyl[D_3_]-methylhistidine appearance in the media, as well as a decay in enrichment from the bound protein pool. In terms of MPB, rates were relatively consistent across time for the initial 24 h post-media change (Fig. 2D), with increases by 48 h. Thus, addition of the methyl[D_3_]-^13^C-methionine tracer provides a novel approach to quantify MPB, through a direct transfer of the methyl[D_3_] group to protein-bound histidine. Moreover, this technique overcomes a major limitation of other MPB tracer techniques in terms of the assumptions made in relation to amino acid recycling from proteolysis, as the methylhistidine is not re-incorporated back into protein, nor further metabolized within the muscle cells.

Having established that the tracer could accurately be used to measure both MPS and MPB rates *in vitro*, further validity for the method was achieved using compounds known to modulate MPS and/or MPB. Both IGF-1 and insulin have both been known to induce anabolic as well as anti-catabolic changes in skeletal muscle cells [[20], [21], [22]], while Dex both inhibits MPS and accelerates catabolism in C2C12 cells [23,24]. The increase in MPS with insulin and IGF-1, and the reduction in MPS with Dex therefore, were consistent with previously reported changes with these treatments, further supporting the use of this tracer as a robust approach for estimating muscle FSR. Accretion of total protein content (Fig. 4A), as well as activation of key anabolic proteins (p70 S6K1, AKT, eEF2) following 24 h IGF-1 and insulin treatments were also indicative of net increases in MPS and decreases in MPB, adding further support for the effects of each treatment.

As with the MPS measures, measuring specific markers of MPB further verified the effects of each treatment. Dex has previously been shown to upregulate muscle atrophy F-box (MAFbx) and muscle RING finger 1 (MuRF1), in line with its established effects on activation of ubiquitin-proteasome pathway (UPP)-mediated proteolysis [25,26]. Conversely, both IGF-1 and insulin treatments have been linked to downregulation of expression of UPP markers MAFbx and MuRF1, at least at the gene level [20,21,27]. Observed decreases in total protein in the present study, as well as decreased activation of anabolic signaling pathways (p70 S6K1, AKT, eEF2), were therefore in line with the expected inhibitory effects of Dex on MPS. In addition, activation of the autophagy marker LC3 (the ratio of LC3-II to LC3-I being a commonly used marker of autophagy [28]) and increased MAFbx protein with Dex (and decrease with insulin treatment), largely confirmed that the treatments were effectively influencing MPB. Thus, the changes in rates of MPB observed following each treatment, i.e. consistent decreases in MPB with chronic IGF-1 and insulin, but elevated MPB following 48 h Dex treatment, are entirely in line with previous studies as described above. These findings provide further support that our method can accurately and reliably be used to quantify changes in myofibrillar MPB in response to known anabolic and catabolic factors. The fact that we were able to detect quantifiable changes in both MPS and MPB across a range of anabolic/catabolic conditions highlights the potential of this novel tracer approach in contributing to our understanding of muscle protein turnover in a wide range of physiological conditions in both cell and pre-clinical models.

In conclusion, in the present study we aimed to overcome some methodological challenges associated with measuring the rates of MPS and MPB within the same experiment, by developing a novel stable isotope technique that utilizes a single multiply labelled amino acid that has the capacity to measure both MPS and MPB within the same experiment. We were able to demonstrate that the tracer methyl[D_3_]-^13^C-methionine could be used for accurately measuring MPS and MPB in a skeletal muscle cell model *in vitro*, using selected treatments previously known to alter MPS and/or MPB to validate the method. We anticipate that with further validation and development this method could be widely used and adapted for a range of physiological settings within the field of musculoskeletal research (particularly in *in vivo* pre-clinical models and potentially in human metabolic research) as well as other disciplines, though this technique may be less suitable for those tissues with low concentrations of actin and myosin. Nonetheless, it is likely that the underpinning theoretical frameworks would be similar across cell types. Ultimately, for the many potential applications possible, the end user would need to optimise our proof-of-concept approach in their cell type/culture systems of interest. Other methylation events downstream of the SAMe pathway could potentially be evaluated with adaptation of this tracer technique, such as DNA or phospholipid methylation, or potentially other amino acids within proteins. Further development and validation will be required to test the potential of this tracer in other cell types/species, particularly aiming to verify that this technique can be applied to studying skeletal muscle protein metabolism *in vivo*.

## Author contributions

DJW, KS & PJA conceived and designed research; HC performed experiments; HC and DJW analysed data; DJW, KS, PJA & HC interpreted data; HC wrote the manuscript; all authors edited the manuscript; all authors approved the final version of the manuscript.

## Declaration of competing interest

None.
